# The Day-to-Day Co-Production of Ageing in Place

**DOI:** 10.1007/s10606-014-9202-5

**Published:** 2014-04-22

**Authors:** Rob Procter, Trisha Greenhalgh, Joe Wherton, Paul Sugarhood, Mark Rouncefield, Sue Hinder

**Affiliations:** 1 Warwick University, Coventry, CV4 7AL UK; 2 Queen Mary University London, 58 Turner Street, London, E1 2AB UK; 3 Barts Health NHS Trust, Newham University Hospital, London, E13 8SL UK; 4 Lancaster University, Lancaster, LA1 4YW UK

**Keywords:** bricolage, co-production, assisted living, ageing in place, telecare, telehealth

## Abstract

We report findings from a study that set out to explore the experience of older people living with assisted living technologies and care services. We find that successful ‘ageing in place’ is socially and collaboratively accomplished – ‘co-produced’ – day-to-day by the efforts of older people, and their formal and informal networks of carers (e.g. family, friends, neighbours). First, we reveal how ‘bricolage’ allows care recipients and family members to customise assisted living technologies to individual needs. We argue that making customisation easier through better design must be part of making assisted living technologies ‘work’. Second, we draw attention to the importance of formal and informal carers establishing and maintaining mutual awareness of the older person’s circumstances day-to-day so they can act in a concerted and coordinated way when problems arise. Unfortunately, neither the design of most current assisted living technologies, nor the ways care services are typically configured, acknowledges these realities of ageing in place. We conclude that rather than more ‘advanced’ technologies, the success of ageing in place programmes will depend on effortful alignments in the technical, organisational and social configuration of support.

## Introduction

Throughout the OECD nations, an ageing population is fuelling interest in assisted living technologies (ALTs) and care services to support ‘ageing in place’ through ‘care at a distance’ (Roberts et al. 2012) – that is, measures to enable older people to live independently at home, avoid or defer institutional care in later life and remain active participants in society (Cohen 2009; Lewin et al. 2010). In response, numerous ALTs and services have been developed and deployed. However, uptake and use has fallen short of levels desired by policymakers (Martin et al. 2008; Vasunilashorn et al. 2012) and there is evidence of significant reluctance to adopt by those who would supposedly benefit (Sanders et al. 2012). Despite this, recent initiatives such as the UK Technology Strategy Board’s £23 M delivering assisted living lifestyles at scale (dallas)^1^ programme aim to accelerate the deployment of ALTs and services.

Our ethnographic research in the ATHENE project^2^ points to a number of reasons for the gap between policy and research enthusiasm (high) and real-world use of ALTs (low). One is a lack of consensus between stakeholders (policy makers, technology suppliers, service providers, groups representing the interests of older people, academic researchers, older people and their ‘informal’ carers) as to what the ‘organising vision’ of ageing in place is – or ought to be (Greenhalgh et al. 2012). However, our studies suggest that even if stakeholders succeed in defining a shared vision for technology-supported ageing in place, its implementation will be compromised unless other, equally pressing, problems are also tackled.

In this paper, we reveal a significant lack of fit between older people’s day-to-day support needs and the technologies and services on offer to meet them (Greenhalgh et al. 2013). We show that the problems that follow from this cannot be resolved without a better understanding of the complex and diverse living experiences and care needs of older people. If the needs of older people are to be met, then technology suppliers and health and social care providers must devote more resources to understanding and supporting ageing in place as it is routinely achieved.

Our investigations reveal in rich detail how ageing in place is socially and collaboratively accomplished – ‘co-produced’ – by the efforts of both formal (e.g. ALT device installers, health and social care departments, telecare call centre workers, sheltered housing staff) and informal (e.g. family, friends, neighbours) networks of care (Bratteteig and Wagner 2013). A key finding is that successful use of ALTs often depends on ‘bricolage’ (pragmatic customisation, combining new with legacy devices) by care recipients’ informal care networks. Unfortunately, neither the design of ALTs, nor the ways that care services are typically configured, acknowledge this critical dependency, making the efforts of informal carers undervalued and of limited effectiveness. As Moreira (2008, p. 102) has observed, “the efficacy of health technologies depends upon users’ work that is largely invisible to professionals, managers and designers”. Our aim in this paper is to recover this invisible work and explore its implications for ALTs and care service design and delivery.

One common technology supplier response to problems with ALTs and services has been to design greater sophistication or ‘intelligence’ into devices such as activity or fall detectors, thereby making them more sensitive to the context in which they are deployed. Such solutions have much in common with the vision of the ‘smart home’, which proposes that technology is the solution to the ‘messiness’ of everyday life (for a critique see Dourish and Bell 2011). Indeed, we find that many of the problems care recipients encounter with ALTs and services lie not in there being insufficient intelligence designed into devices or for the want of a ‘smart home’, but from the problems care providers experience in mobilising the intelligence and skills in the social network (family, friends and neighbours) in which the older person is typically embedded (Roberts et al. 2012).

In summary, we argue that the ‘lived reality’ of ageing in place is currently not adequately acknowledged in the design of ALTs or in the configuration of the associated care services. Though our evidence is UK specific, other studies suggest that this is true of other countries with similar demographic and socio-economic features (Breskovic et al. 2013). If this increasingly global problem is to be addressed, then technology suppliers and service providers must develop ways of supporting ageing in place as experienced by older people and their informal carers. In this paper, we explore the issues and report on research that suggests possible ways of moving towards a more inclusive, co-production approach.

## The ATHENE project

The ATHENE (Assistive Technologies for Healthy Living in Elders: Needs Assessment by Ethnography) project (Greenhalgh et al. 2011) was funded by the Technology Strategy Board under its Assisted Living Innovation Platform programme.^3^ The project sought to produce a rich understanding of the lived experiences and needs of older people, and explore how stakeholders – suppliers, health and social care providers – can work with care recipients and carers to ‘co-produce’ ALT and service solutions.

The project consisted of two phases. Phase one involved interviews with ALT suppliers and service providers. This was followed by detailed ethnographic studies of 40 individual cases in and around the person’s home to map the complex healthcare, social care and socio-cultural needs of older people and their carers from a range of ethnic and social groups. Phase two took forward exemplar cases and using participatory design methods to explore how older people and their families can work directly with industry designers to produce fit-for-purpose technologies, or adapt existing technologies, that fit in with people’s lives and lifestyles.

## Adoption issues for assisted living technologies and services

Assisted living technologies comprise the sensors, devices and communication systems that, in combination, support delivery of services to a person in their own home. They include telehealth (remote medical care, treatment or monitoring) and telecare (remote social care services or monitoring), proposed as a solution to the inter-related trends of ageing of the baby boom generation; rising rates of chronic illness and disability; shortfalls in health system capacity and budgets; and shifting social roles and expectations (Cohen 2009; Lewin et al. 2010).

Ageing in place support covers a diverse range of technologies and care services that enable remote monitoring (e.g. blood pressure and blood sugar levels) or prompting of individuals (e.g. falls detection, room occupancy, location of wanderers, medication reminders) and/or homes (e.g. detection of smoke, heat, gas, overflowing baths, medication not taken and unlocked doors). Of the 1.7 million installations in the UK, all but 300,000 are pendant alarms (Clark and Goodwin 2010). These are generally linked with local social services departments or call centres, which assume some level of responsibility to interpret and respond to these signals. Care recipients may be charged for the technology and/or the service that is supported by it. With advances in Internet and mobile technologies, there has also been growing interest in alleviating loneliness of older people by supporting social connectivity (e.g. Wherton and Prendergast 2009), though some have expressed concerns that this might lead to less traditional face-to-face care and decreased social contact (Milligan et al. 2011). For example, the EU Ambient Assisted Living (AAL) Joint Programme,^4^ launched in 2009, focused on developing *“*ICT based solutions for advancement of social interaction of the elderly”. Nevertheless, large-scale efforts to promote the adoption of ALTs, such as the UK dallas programme, have mainly focused on addressing the physical challenges of ageing in place.

In order to establish current perceptions of the challenges ALT suppliers and service providers face in designing and deploying effective solutions to care recipients’ needs, we carried out semi-structured interviews with people involved in the development of ALT devices and provision of services in the UK (for a full account see Sugarhood et al. 2014).

Interviewees confirmed that the appearance and style of many devices were off-putting, representing the medical world of instruments and monitoring, rather than the personal world of family and culture, and carried stigma attached to old age, illness and disability (Lehoux et al. 2004). They also noted that care recipients and their informal carers often had to alter their daily routines to fit in with service requirements (e.g. providing blood pressure measurements at a fixed time in the day).

Interviewees reported that care recipients frequently used ALT devices and services in ‘wrong’ ways. For example, some would press the alarm button to report that their carer had not arrived, or for social contact when lonely or anxious. Interviewees acknowledged that such cases of care recipient ‘reinvention’ ought to be reflected in ongoing efforts to adapt technologies and services to actual requirements.

The current generation of devices can be set up to connect with 24-hour call centres, family members or some combination of these. For example, call centre staff may have an arrangement with the care recipient’s family as to who will be called, under what circumstances and what actions will be taken when an alarm is triggered (Sugarhood et al. in preparation). Interviewees agreed that this network of formal and informal carers – the ALT ‘soft periphery’ – must be adaptable to the needs of the care recipient (Denis et al. 2002), but reported that this did not always occur in practice.

Many ALT device suppliers are trying hard to involve care recipients in the design and development process, from initial concept through to final product. Methods used include design workshops, installing prototype devices in individuals’ homes and forums to gather feedback on products. Interviewees suggested that such ‘user-centred design’ tends to focus on ensuring technical usability and proof of concept of a specific device, rather than on considering the technology in the context of the care recipient’s wider needs or its impact on their life more generally.

Interviewees reported that care recipients often have an opportunity to gain some initial hands-on experience of ALT devices, e.g. a show flat or a demonstration area attached to a day centre for older people. However, the adoption decision was often not the care recipient’s alone, but contingent on the views of informal carers. As importantly, adoption was not an all-or-nothing, one-off event. Rather, it was a process that evolved over several months or years as care recipients and their carers gained experience of living with the technology.

Poor usability has often been cited as a key reason for the low acceptance of the first generation of telehealth and telecare devices (Lehoux 2004; Lehoux et al. 2004; Gately et al. 2008). So, it is encouraging that ALT suppliers and service providers reported considerable progress in addressing basic issues of ALT acceptability and usability. However, it is clear from our fieldwork, which we report below, that the realities of how ageing in place is accomplished day-to-day are not adequately acknowledged in either device design or care service provision.

## Methodology

We conducted fieldwork focusing on developing an understanding of older people’s lives and their experiences of ALTs and services. Forty older people, aged 60–98 (recruited via UK NHS and third sector organisations), were visited at home several times. Using ethnographic methods, including cultural probes and interviews, we built a detailed picture of their lives, illness experiences and use (or non-use) of ALTs and services (Wherton et al. 2012). Subsequently, we hosted workshops to provide opportunities for participants to share experiences (Wherton et al. in preparation).

Pursuing ethnography in domestic settings raises practical and ethical challenges. Cultural probes offer a relatively unobtrusive way of providing insight into how technology could fit (and why it sometimes does not fit) into a particular home environment. Probes have variously been described as: “Collections of evocative tasks meant to elicit inspirational responses from people” (Gaver et al. 2004, p. 53); “materials…treated as resources facilitating cooperative analysis” (Crabtree et al. 2003, p. 8); “an automatic recording device that is sent to unknown territories where human researchers cannot go, from where it collects samples, and sends these back to the researchers” (Mattelmäki and Battarbee 2002, p. 266). The method includes open-ended and evocative activities for participants to pursue in their own time to help narrate and depict their lives to researchers and technology designers. It uses digital cameras, dictaphones, diaries and other artefacts to capture aspects of people’s everyday lives, their problems and aspirations (Gaver et al. 1999) and promote and encourage participation, ensuring that people are active participants in the research process.

Cultural probes have been used fairly extensively in design research and have begun to be applied in domestic settings for non health-related design projects where access for conventional observational study methods is problematic. Gaver et al. (1999) used probes as inspirational tools for design teams and their value was considered to lie in the uncertainty and subjective interpretation of the materials produced. The intention was not to identify a specific set of problems or technological requirements, as would occur in a formal design specification but, instead, to capture in a more general way users’ “beliefs and desires, their aesthetic preferences and cultural concerns” (Gaver et al. 1999, p. 25). Others have used cultural probe methods to help build a rich understanding of participants’ perspectives and experiences (Graham et al. 2007). The latter approach informed our own application of cultural probes, with the aim of using them as a tool to promote dialogue between older people and project team members.

For the ATHENE project, we developed a version of the cultural probe, which we named the ‘Home and Life Scrapbook’ (Table 1). This consisted of an A4 booklet (Arial font size 18) containing seven activities to help capture information on physical, emotional, social and environmental factors related to health and independence at home (Wherton et al. 2012).

**Table 1:** Summary of the home and life scrapbook activities.

Activity	Description
Camera	Digital camera to take photos during the week
Maps	Drawings to show relationships with people, places and objects
Lists	Lists of what they like/dislike, what concerns them and what they are comfortable with.
Wishes	Three things they would like to improve or change about their lives
Body outline	Drawing onto a body outline to indicate symptoms or impairments (e.g. pain, discomfort, weakness or decline)
Home plan	Room layouts to indicate spaces and objects related to daily routines and health
Diary	Activities and events they choose to record over 1 week

Each participant was visited on at least three occasions. On the first visit, we explained the purpose of the project and asked the participant to consider taking part; we left an information sheet with them. On the second visit, we conducted a semi-structured interview focusing on routines, health, social networks and technology use. At the end of the interview, we presented the Home and Life Scrapbook and camera and went through each suggested activity in turn, emphasising that they could choose which, if any, to complete. On the third visit (approximately 1 week later) the researcher and participant reviewed and discussed the digital photos and scrapbook content together. Following the interview, we conducted a ‘home tour’, in which the participant showed us different areas of their home to prompt further discussion about what they did and problems they faced.

To situate this study of older people’s daily needs within the wider network of support and service provision, we also carried out observations of three call response centres. Two were telecare call centres and one a combined telecare/telehealth call centre. The aims were to understand the kinds of issues callers have, how call takers resolve them and the resources they call upon to do this. We spent up to 6 hours in each centre listening in to the calls and talking with staff about how the systems operate and how they make decisions (for detailed findings see Sugarhood et al. in preparation).

## Findings

Each participant in our study had multiple, mutually reinforcing impairments (e.g. tremor *and* visual loss *and* stiff hands) that were culturally framed (as reflected, for example, in ethnicity, familial bonds and geography), steadily worsening, and bound up with the prospect of decline and death. We found that installed assisted living devices met relatively few of participants’ needs; some had been abandoned and a few deliberately disabled.

We provide below a series of selected extracts from fieldwork records of participants’ experiences of ALTs. They illustrate how successful use often depended on ‘bricolage’ (pragmatic customisation, combining new with legacy devices) of the devices by care recipients’ informal support networks, including their family members or neighbours. The names of participants have been anonymised.

Bonnie, aged 81, lives alone in a bungalow close to her daughter Carol. She has multiple health conditions including chronic obstructive pulmonary disease (COPD), heart disease, visual loss, anxiety and type 2 diabetes. Her home is fitted with both telehealth and telecare devices which she can’t use without the help of her daughter. The telehealth unit consists of an oximeter, thermometer, weighing scales and a blood pressure monitor. Carol calls in twice a day. She helps her mother to wash and dress, makes all the meals and takes her out regularly.

At 10 am a voice comes from the telehealth monitor “Please take your daily measurements”. This is Carol’s cue to do the morning routine of medications and monitoring, not just the telehealth monitoring.

Carol prepares the insulin pen the night before in case something happens and she can’t get there in the morning. Bonnie can’t see well enough to prepare the pen herself.

Carol then gets 2 dosette boxes, there are too many tablets for one box. She says her mother couldn’t open them by herself, they are too fiddly, so Carol transfers that day’s medication into a 1 day box, which is easier to open.

The scales are under Bonnie’s chair and Carol gets them out and helps her mother on to them. Carol says it’s difficult for older people to balance on scales. Carol puts the BP cuff on, but has to strip off Bonnie’s dressing gown and pyjamas. Bonnie would not be able to do the BP and the weighing scales herself.

After that, Carol prepares her mother’s lunch and leaves it ready for her to microwave. She leaves the food actually in the microwave with the correct settings so that all Bonnie has to do is close the door – usually soup or a bowl of rice pudding. She makes Bonnie another hot chocolate before she leaves.

Bonnie mentions that her neighbour next door hears she’s ill, through hearing her coughing, and she will ring Carol.

Bonnie sometimes gets confused and thinks there is someone in the house, She rings the Police for assistance, but the Police know Bonnie and they ring Carol to tell her what’s happening and she goes round to Bonnie to reassure her.

Fieldwork Extract 1: Bonnie

Bonnie’s daughter Carol is a skilled ‘bricoleur’, applying her intimate knowledge of Bonnie’s desires, needs and physical limitations to obtain and/or adapt technologies and daily routines to make maximum use of Bonnie’s remaining capacity (Fieldwork Extract 1). Bonnie relies on her to provide workarounds to help her cope with equipment that is too difficult for her to use. A neighbour is on hand to alert Carol to possible health problems, and the Police and Carol have worked out a routine to deal with Bonnie’s episodes of confusion.

Nadine is a 90-year-old widow who has just returned home after a stroke and about 3 months as an in-patient on a hyper acute stroke unit and then rehabilitation unit. She has pull cords in her sheltered flat but these are out of reach. There is a red button on the control unit, which is located very close to the front door. This frequently goes off when carers and others enter the house and brush past it. Her son has taped a jam jar lid over the button to prevent this happening.

He was negotiating with the Housing Association about installation of alternative telecare, e.g. a pendant alarm. He would also like heat and carbon monoxide sensors but has been told that these are not available. He believes Nadine may struggle to adapt to and learn to use these devices even after much training, which will have to be done by him/the family.

The warden – who has no technical background – visited last week to install the device. She brought the control unit and a pendant alarm, but could not connect the existing telephone into the control unit as she did not have the correct lead. Nadine’s son says she is due to return shortly to complete the job, but has no confidence this will happen.

Nadine’s son has implemented some successful technological adaptations, e.g. purchasing a microwave and toaster, which Nadine is able to use to maintain some independence with meal preparation. He is now thinking of installing a hot water dispenser to enable Nadine to make hot drinks in the kitchen, as she cannot use the kettle. There might be cheaper and safer alternatives such as a kettle tipper, but he is not aware of them.

Fieldwork Extract 2: Nadine

**Figure 1:**
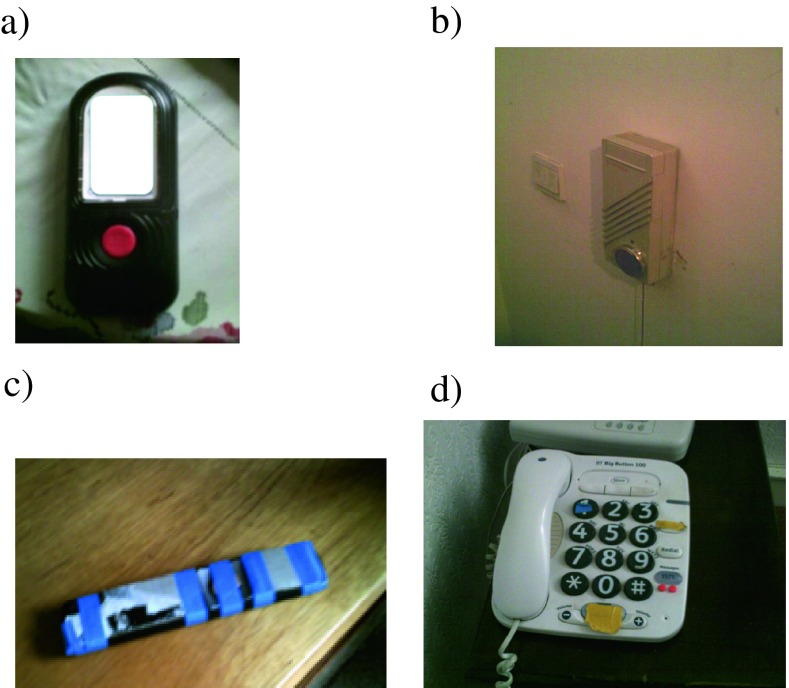
Examples of ad-hoc adaptations of domestic equipment. **a** Phone numbers of family members written on paper and stuck on the back of a mobile phone as this was perceived as easier than storing and accessing numbers in electronic phonebook feature; **b**) Jam-jar lid covering the button on the call unit to prevent accidental setting off of alarm (Fieldwork Extract 2); **c**) Tape covering buttons on TV remote so that only the channel and on/off buttons are exposed, making it easier to use with visual impairment (Fieldwork Extract 5); **d**) Phone with post-it notes and raised dots stuck onto it so the user, who has severe visual impairment, can feel their way around it.

Nadine’s son acts as both her advocate for telecare installation and as adapter of technologies that she is no longer able to use in their standard form (Fieldwork Extract 2). One of his adaptations has been to Sellotape a jam jar lid over the call unit to prevent its accidental triggering of the alarm (Figure 1b). Even though various statutory health and social agencies are involved in Nadine’s care, her informal care network plays a vital role to ensure her day-to-day needs are met. However, her son appears to lack information about commercially available adaptations of domestic appliances that might avoid him having to fashion more ad hoc solutions to his mother’s needs.

Bilal, a 70-year-old man originally from Pakistan, lives alone in a ground floor flat. He was leading an active retirement until about 1 year ago when he had a severe stroke, which dramatically changed his life. He is now unable to go outdoors alone. Either his brother or one of his nephews or nieces visits daily during the afternoon to prepare meals and sometimes support Bilal to take a short walk around his block of flats.

Since returning home from the stroke unit, he has had a landline phone and broadband connection installed. Two of his nephews work in IT; they organised the connection, set up a Gmail account, taught Bilal how to use his iPad, laptop and Skype, and are available to fix any problems. His iPad was purchased by a friend and given to him while he was on the stroke unit. He has hundreds of apps on it and appears to use them competently; many are games or for drawing, and he also has exercise video apps for his arms and legs. He stays online 24 hours a day so he can call and receive video calls from his family (including his children) in Pakistan.

He was a mobile phone user pre-stroke, and had to install a landline in order to get a telecare service. It was at this point that his family thought of broadband.

There are stand-alone environmental sensors in the flat (smoke, heat, carbon monoxide), provided over the years due to various refurbishments or schemes in the (council) block. Bilal was not involved in the decision to install these devices. No attempts have been made to link them in with the telecare system.

What really matters to Bilal, however, is walking outdoors and getting out, connecting with the places he frequented prior to the stroke. The pendant alarm only works indoors and he does not feel safe going out alone.

Fieldwork Extract 3: Bilal

Bilal’s access to useful technologies is facilitated by his nephews’ expertise in IT (Fieldwork Extract 3). Yet, even they are unable to deal with two deficiencies that limit their effectiveness. The first is the lack of integration of the various individual devices and the second is the limited range of his alarm. It appears that no solutions to these problems are on the horizon.

Molly, aged 77, lives alone in a 3-bedroom house. She was diagnosed with macular degeneration 2 years ago. At the time of diagnosis the doctor said she would be blind in a few months but in fact she was blind within weeks. She describes herself as “depressed” since.

“It’s a shame I’ve got that lovely computer upstairs and I can’t use it.” The family bought it for her 7 years ago. She used it a lot before for writing stories, writing to pen friends abroad and for getting information. She thought it was really great and she really misses it. She used computers all the time for accounts when she was at work; she found it ‘a doddle’, but now she can’t use the computer and she, and her son are unaware that there are computer programs that would allow her to use a computer again.

Molly points out the TV remote control. Her son, Gary, has covered it with cardboard and blue tape to make sure she can only use the channel changer and volume buttons. In the past she has pressed lots of buttons trying to change channels and ended up not being able to get the TV back on. In the past she has sat there with no TV and no radio and no CD player because she’s messed up all the control buttons. Gary says he has also put the radio programmes through the TV so there is one less button to press. With the remote he has covered over all of the buttons apart from the channel changer and the volume.

How does she manage the tumble dryer? She knows where the dials are but she forgets from week to week. She fiddles with the dials until the dryer comes on. She says she’s not very good with dials and buttons probably because she doesn’t press hard enough. She gets the dryer going eventually but she says it’s ‘practising with it’.

There’s lots of blue tape over the buttons on the washing machine. She says she thinks this is because she would be pressing the wrong buttons otherwise. She puts her fingers over the washing machine control panel. She’s not sure what the buttons are. It’s set for a cold wash, so she washes everything on a cold wash. Gary’s done that since she’s gone blind so that she can put different clothes in together.

Fieldwork Extract 4: Molly

Molly’s domestic technologies have been extensively, if simply, adapted by her son so she can cope with her failing sight and memory (Fieldwork Extract 4). For example, he has used tape to mask some of the TV remote control buttons (see Figure 1c). However, her lack of awareness of, and capacity to use, computer programs for visually impaired people (even though she has had contact with a voluntary organisation for the visually impaired) denies her a source of pleasurable activity and inhibits her contact with the outside world. This last point illustrates the severely limiting effect when there is no bricolage or the bricoleur does not have the necessary knowledge and skills to make the required adaptations.

We see five recurrent themes in these fieldwork extracts of older people’s experiences with ALTs. First, the importance of informal carers for ensuring day-to-day care needs are met. Most of the study participants benefit from some level of support from family and/or neighbours, which helps them to manage. Where these are not available, we find the individual’s capacity to cope is diminished.

Second, these informal carers are typically linked in networks that vary widely in their extent and composition. Some networks are limited to close family members, others involve neighbours or other people recruited by family members. The requirements for membership of a care network seem to be a willingness to be involved, a useful skill and trustworthiness. In some cases, we found the existence and significance of these informal networks is acknowledged by formal care providers but, in others, it is not.

Third, one of the key roles members of these informal networks play is of adapter or ‘bricoleur’ of the technological devices on which the care recipients rely for their day-to-day security and wellbeing. Our examples illustrate how ordinary domestic equipment and ALT devices are often more effective when there is someone on hand to improvise, tinker with and customise them to the person’s needs.

Fourth, the adaptations are sometimes crude (e.g. the disabling of a call unit button, see Figure 1b; use of tape to make certain buttons on a remote control inoperable, see Figure 1c), a consequence, unquestionably, of the lack of ‘designed-in’ configurability of many of the devices and the uniqueness of individuals’ needs. Many of the adaptations we observed were small but crucial. Bricolage – ‘design in use’ (Procter and Williams 1996) – bridges the design-reality gap in ways that are sometimes very subtle.

Fifth, homes are hugely varied, both materially and culturally – and in reality, many are cluttered, cramped, old, in poor repair and a far cry from the futuristic and spacious ‘smart home’ of the designer imagination (Dourish and Bell 2011). Bricolage adapts the technology not only to the person’s physical and cognitive capabilities but also to the material and cultural constraints of the home.

## Discussion

“*For every spectacular failure of infrastructure there are hundreds or thousands of small everyday niggles and petty failures – doors that jam, mobile phones that need to be power cycled, keys that require a special touch, antennas that need to be aligned just so, screws that refuse to turn, wireless networks with inadequate coverage, operating systems that lose track of memory, plumbing that backs up, or cables that need to be jiggled. Many of the indignities of everyday life revolve around the continual mutual alignment of individual action and infrastructures that don’t quite support it, either because they do not operate entirely as advertised or because they have been pressed into service to fulfil needs that they were never intended to support.”* (Dourish and Bell 2011, p. 113)

We see in these fieldwork extracts how ageing in place is collaboratively realised, co-produced by the efforts of a range of informal carers. This is despite the evident lack of affordances designed into the ALT devices for customisability and configurability that would assist informal carers in adapting them to care recipients’ needs.

Bricolage originally referred to making do with tools that are available to address an immediate, local and contingent problem or need. Kirmayer emphasised how the bricoleur, who “thinks with things to create an order based on the logic of the concrete”, is applying knowledge that is practical and opportunistic rather than theoretical and abstracted (Kirmayer 1993). In the context of technological artefacts, bricolage emphasises crafting solutions using whatever is at hand, “the rapid assembly and configuration of ‘bits and pieces’ of software and hardware” (Hartswood et al. 2000, p. 2), blending new and second-hand materials to produce one-off devices and adaptations for one-off problems (Büscher et al. 2001).

Bricolage can be understood as a normal, natural response to the failings of a priori design of technology and its limited horizon for anticipating users’ requirements, both initially and as they evolve over time (Hartswood et al. 2002, 2008). Its role for ensuring the effectiveness of information and communication technologies (ICTs) in work environments has been extensively studied, for example, the fashioning of ‘work arounds’ in the work place in order to expedite processes or deal with technical failures (e.g. Ciborra and Lanzara 1994; Shapiro et al. 1996; Hartswood et al. 2000; Voss et al. 2000; Büscher et al. 2001, 2002; Büscher and Cruickshank 2009). However, its role in the domestic arena has been less studied (Blackwell 2006). Our evidence points to its importance in this context but we need a better understanding of its contribution and scope – its limits as well as its possibilities – if ALTs are going to be useful and usable. Bricolage emphasises that users (in this case care recipients, family members and friends) will often resist being ‘configured’ (Woolgar 1991) and actively attempt to shape the meanings and uses of technologies (Lehoux et al. 2004).

Facilitating bricolage for ALTs raises a number of issues. First, although it might be understood as a pragmatic response to the failures of conventional, a priori design, there are ways in which designers can respond. If assisted living devices were designed to permit a degree of customisation, then this will make them more easily adaptable to each person’s needs. Our study suggests, however, it is also important that individual devices are capable of being assembled into larger configurations (see Field Extract 4). This would enable, for example, different monitoring devices (e.g. for blood pressure, weight, etc.) to integrate with a single unit for the transmission of readings. Moreover, such a configuration could be adapted (e.g. with the addition of new devices) as the person’s needs change. What this example highlights is the benefits of designing ALT devices as composable units that enable the construction of more complex and bespoke solutions in simple and straightforward ways (Cabitza and Simone 2012).

To realise this composability, individual ALT devices must adhere to common standards for data interchange and control protocols (Cabitza and Simone 2012). In the UK, however, despite the efforts of the Continua Alliance^5^ to promote standards for interoperability among technology suppliers, progress towards this goal is reported to be slow. Such a fundamental reconfiguration of the mode of technology supply may take time to achieve and is likely to be resisted by where suppliers’ business models rely on ‘locking’ users into their products.

An alternative way forward would be to explore how technologies from other domains could be repurposed to support ageing in place. Mobile computing devices (smart phones and tablets) and the ecosystem of software components (‘apps’) that has rapidly emerged around them have had a significant impact on people’s relationship with technology. Where once the majority of computer users had to be content with whatever software was pre-installed, customising mobile devices by adding apps is now a familiar and routine activity for many. One key to success has been the diversity in the supply of apps which, in turn, has been greatly facilitated by the availability of software environments that enjoy de facto standard status: Apple iOS, Google Android and Microsoft Windows. Could one or more of these provide a platform for a new generation of assisted living technologies? Emerging social media platforms such as Facebook might also be re-purposed, especially as they are explicitly designed around a model of shared communication.

Second, in the context of ageing in place, it is essential to consider the safety risks that increased scope for customisation and composability of ALTs may create, and how these may be addressed. Given the lack of support for customising currently designed into ALT devices (Rogers et al. 2011), it is not surprising that the response of some participants in our study was simply to turn devices off, with potentially unsafe results. However, designing customisability into ALT devices may also increase safety risks and these may escalate where multiple options and/or devices are involved. From a technical perspective, to what extent can safety and dependability be guaranteed by design? If design is only part of the solution then what other measures might be put in place. In particular, from the perspective of the customiser, what skills might be required, how much familiarity with the care recipient and their situation and how might these requirements can be satisfied by the expertise within their formal and informal care network?

Third, and following on from the above, customising ALTs in a dependable way requires that we view ALTs as elements of collaborative networks, tying patients, technology suppliers, family and informal carers, health and care service providers – the soft periphery of ALTs – together. Customisation must therefore be understood and measures taken to support it as a collaborative activity, as one element of the wider work of co-producing ageing in place, where effective and dependable support is realised through the collective efforts of all those involved, so that solutions can be scrutinised and risks minimised. Designing to support collaboration affords more dependable and safe systems (Procter et al. 2006; Voss et al. 2006; Fitzpatrick and Ellingsen 2012).

These findings collectively point to the need to consider the role of collaboration in the successful accomplishment of ageing in place. Our studies of the work of telecare call centres show that collaboration plays an important part in managing problems (Sugarhood et al. in preparation). For example, details of the care recipient, their habits and routines, and the members of their support network are usually displayed on the call centre worker’s^6^ screen when a call is received. Resolving the call quickly and successfully depends on the call taker having access to information about the care recipient in a timely way. Through the telecare call centre IT system, the call taker is able to see a log of the care recipient’s previous calls, a summary of their medical condition(s), the services they receive and the people involved in their care (both formally and informally). Hence, in principle, the call taker is in a position to ‘join up’ what otherwise are often fragmented services. However, in practice, the work of ‘joining up’ can often entail a lengthy and not always successful process of ringing around different people (e.g. family members) in an attempt to ‘fill in’ information about a care recipient’s current status not routinely captured by the system. As the following example from our observations of telecare call centre work illustrates, for all the sophistication of the technologies, it is the tacit knowledge and persistence of the workers that holds the operation together (Sugarhood et al. in preparation).

Mrs Jones, who used a telehealth system to monitor her heart condition, suddenly stopped sending her blood pressure and weight readings. It took some time for staff in the monitoring service to find out that the reason was she had been admitted to hospital.

Several telephone calls with Mrs Jones’ family (who lived some distance away) ensued to establish when the patient would return home. Following discharge, telehealth readings were still not received, however, as the patient was no longer able to get upstairs where all the devices were located.

Time-consuming liaison with Mrs Jones and her informal care network was required to discover what had happened and decide what course of action to take. Would Mrs Jones regain sufficient mobility to use the stairs? Did she need referral to the technology supplier to bring the equipment downstairs, or to the local authority for a social care package?

Telecare call centre work (extract from fieldwork).

This example draws our attention to a broader and more complex set of problems that derive from how existing IT systems and governance regulations structure the ways in which information is produced and shared within the network of carers and which militate against making decisions in a timely and effective way. Some of these problems arise from a lack of interoperability between different IT systems, in this case the telecare and hospital IT system. In another case, one call centre we observed uses GPS tracking devices for people with dementia. However, the IT system is separate from the main telecare system, making it difficult for the telecare worker to see a full picture of the care recipient’s status. Other problems arise from information access policies that fail to acknowledge the value of knowledge held by informal carers, such that the only way the telecare worker can obtain it is to phone around. A solution would be to reconfigure IT systems that currently function as ‘silos’ of information as shareable information spaces that all relevant parties may access and update (with suitable access policies to protect confidentiality), thereby helping to maintain better awareness within the wider care network of the care recipient’s circumstances day-to-day and enable more effective decision-making and coordination of responses to events.

## Taking the co-production of ageing in place forward

Our findings make a compelling case that successful support for ageing in place depends on making better use of the contributions of all participants in the care network if it is to deliver its promised benefits to older people, and to the health and social care system as a whole. Participants, whether they be the care recipient, their formal or informal carers or technology suppliers, must be able to work together to shape technologies and services, evolving and sometimes even reinventing them over time to meet the needs of the individual.

One of the barriers to achieving this is the absence of a technical infrastructure capable of facilitating the pooling of knowledge that is held within the care network – but not currently easily shared – of the circumstances and needs of the care recipient, to share expertise in problem solving, the configuration of devices and the management of their needs and, most importantly, providing the means to build and sustain relationships between care recipients, their formal and informal carers. Hence, we argue that efforts to improve people’s capacity to age in place should focus not on a narrow vision of making ALTs more intelligent or ‘smarter’ homes, but on ways of mobilising the knowledge and intelligence in these care networks (Roberts et al. 2012).

**Figure 2:**
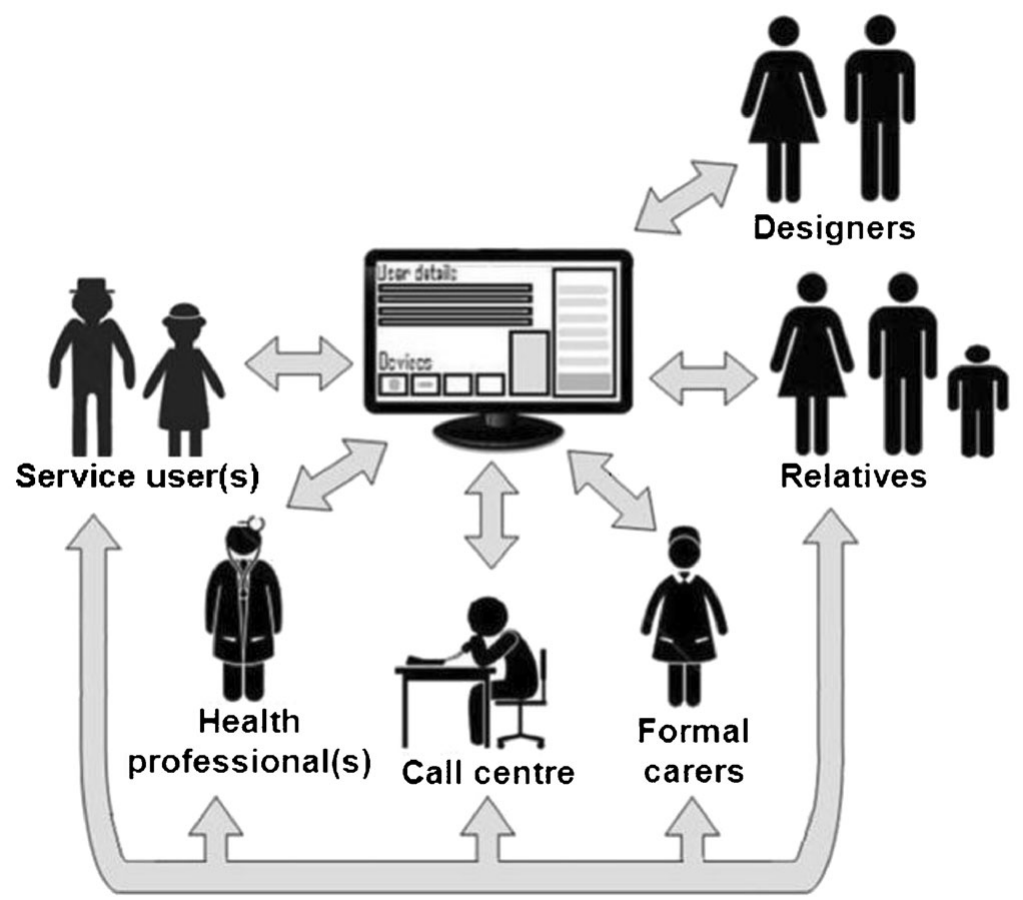
Ageing in place shared ‘dashboard’ supporting collaborative configuration of devices, mutual awareness of unfolding events, monitoring and interpretation of data such as blood pressure, blood sugar readings, physical activity.

A significant step forward would be to provide collaborative interfaces to ALT devices and services and secure, managed information spaces – a shared platform or ‘dashboard’ – supported by multi-modal access mechanisms (e.g. text, voice, video), to enhance opportunities for formal and informal carers to work together (see Figure 2). This dashboard – a ‘Facebook for ageing in place’ – would provide the means to maintain mutual awareness by sharing information, log the use of and collaboratively manage the customisation, integration and configuration of different devices and so support the co-production of ageing in place by the key actors – i.e. the care recipient, their formal and informal carers, and technology suppliers.^7^ In this way, for example, the time consuming ‘liaison work’ evident in the telecare fieldwork extract may be avoided.

We have presented the dashboard concept at a workshop for technology suppliers, formal and informal carers. Suggestions from participants for requirements and how it could support ageing in place included: enabling mutual awareness between formal and informal carers day-to-day of events and changing circumstances (e.g. calendar, medication, reminders); using this shared awareness to assist with the monitoring and reliable interpretation of data (e.g. blood pressure, blood sugar readings, physical activity); providing suppliers of ALT devices with access to a detailed record of how they are being used (including configuration) to enable the identification of problems; supporting the safe (re-) configuration of devices for individual needs; and informing design. In summary, the consensus was that the dashboard would help make possible grounded, collective decision-making about how to deal with problems and support the evolution of ALTs and services for ageing in place (Wherton et al. in preparation).

In these ways, we argue it will be possible to mobilise the knowledge and expertise of all those who can contribute to making ageing in place work to co-produce solutions that will meet people’s needs and will continue to do so over time, even as these needs change. Of course, the dashboard must be secure against unauthorised access and improper use if patient rights are going to be protected. It must also be recognised that governance frameworks setting out how patient information may be shared may present a significant barrier to the realisation of the dashboard’s potential. In the UK, for example, inter-agency working is hampered by perceived difficulties in sharing patient information between health and social care (Richardson and Asthana 2006). Our workshop participants were unanimous that this has to change and a recent review of UK patient information governance principles suggests that the need for change is now accepted (see Taylor 2013 for a brief summary).

**Figure 3:**
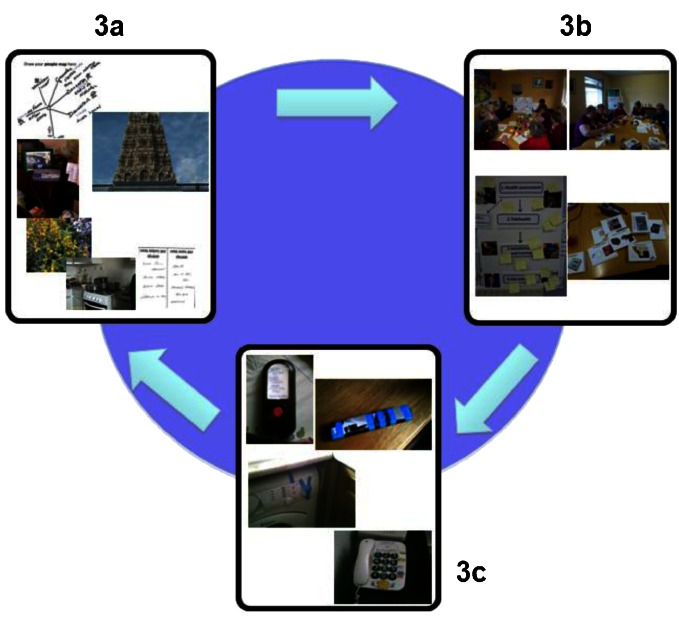
The co-production cycle – tracking the evolving relationship between ALTs and services and the lived realities of ageing in place. **a**) grounding requirements through ethnography e.g. home visits and cultural probes; **b**) involving older people in co-design activities such as workshops; **c**) monitoring and evaluating the use of devices and services.

Finally, we stress that better technical solutions for supporting ageing in place must be underpinned by technology and service stakeholders committing to following a robustly user-centred approach to device design and service delivery. We recommend that this should be based on an iterative cycle consisting of: grounding requirements through domestic ethnography, e.g. home visits and cultural probes (Figure 3a); co-design activities with care recipients and their informal carers (Figure 3b); continuous monitoring and evaluation of the ways in which devices and services are actually used (Figure 3c). Making use of participatory design methodologies such as those employed in the ATHENE project, e.g. cultural probes (Wherton et al. 2012) and co-design workshops (Procter et al. 2013), is basic. At the same time, it is important that technology suppliers and service providers have the capacity to track the evolving relationship between devices, services and care recipients’ needs and feed user experience back into device design and service configuration. This information would assist, for example, in the planning of subsequent domestic ethnographies. Subject to appropriate data privacy policies, by providing a ‘window’ into the day-to-day realities of ageing in place, the dashboard may also play a key role in supporting a user-centred approach.

## Conclusions

*“… our contemporary technological environments do not arrive all as a piece but rather are cobbled together bit by bit, each element pressed into service alongside the others…through a process of bricolage.”* (Dourish and Bell 2011, p. 207)

Our investigations have revealed how ageing in place is collaboratively accomplished – ‘co-produced’ – by the efforts of both formal and informal networks of carers and older people themselves. They have reinforced evidence from earlier interviews with ALT suppliers and care providers (Sugarhood et al. 2014) that major changes to systems, structures and ways of working will be necessary if the vision of ageing in place is to be realised safely and cost-effectively. Assisted living stakeholders need to rethink how ageing in place is technically, organisationally and socially configured and, in particular, examine how they might afford the more effective involvement of care recipients and their networks of informal carers in co-production processes.

We have argued that if ALTs and services are to be fit for purpose, their design and deployment must be grounded in care recipients’ lived experience. This is not currently being achieved, as is demonstrated by our findings of how bricolage is a common response for ensuring that ALTs satisfy the needs of their users. Bricolage is important because it fills the gap between the limitations of a priori design and the lived realities of ageing in place by enabling care recipients and their networks of carers to take the initiative in customising devices to meet their needs. We have examined how new models of technology supply, such as ALT apps, can contribute to making customising and configuration more straightforward and effective and discussed some of the challenges of realising customisable and configurable solutions in a safe and dependable way. We have also made a case for how domestic ethnography and co-design workshops can improve understanding of care recipients’ needs and make design processes more inclusive.

We conclude that the way to address these and wider issues relating to facilitating the co-production of ageing in place is to provide better support for the routine collaboration between members of formal and informal care networks and we have outlined the shared dashboard concept as a means to achieving this. However, there are significant technical and organisational challenges to realising this concept in a practical, secure, dependable and cost-effective way, and these will need to be addressed.

Further work is planned in two specific areas. First, we will continue to refine our methodologies in order to reduce barriers (e.g. social inhibition, frailty) to the participation of older people in co-design activities. Second, we aim to explore in greater detail the potential role for a shared dashboard as an environment for supporting ageing in place.
